# Unveiling the Immunostimulatory Potential of Rhus Toxicodendron in Immunocompromised Balb/C Mice Induced with Cyclophosphamide

**DOI:** 10.3390/diseases12080178

**Published:** 2024-08-08

**Authors:** Vara Prasad Saka, Narasimha Kumar G. V., Bharat Kumar Reddy Sanapalli, Abanti Goswami, Anirban Roy, Anurag Agrawal, Pankaj Gupta, Digvijay Verma, Subhash Kaushik

**Affiliations:** 1 Department of Pharmacology, Drug Standardization, Dr. Anjali Chatterji Regional Research Institute for Homeopathy, Under Central Council for Research in Homeopathy, Ministry of AYUSH, Kolkata 700035, West Bengal, India; 2 Department of Pharmacology, School of Pharmacy and Technology Management, SVKM’s Narsee Monjee Institute of Management Studies (NMIMS) Deemed-to-be-University, Jadcherla 509301, Hyderabad, India; 3 Virology Laboratory, Dr. Anjali Chatterji Regional Research Institute for Homeopathy, Rajendra Chatterjee Road, Kolkata 700035, West Bengal, India; 4 Department of Pharmacology, Ram-Eesh Institute of Vocational and Technical Education, Greater Noida 201310, Uttar Pradesh, India; 5 Department of Pharmacology, Drug Standardization, Dr. D P Rastogi Central Research Institute for Homeopathy, Under Central Council for Research in Homeopathy, Ministry of AYUSH, Noida 201301, Uttar Pradesh, India; 6 Drug Standardization, Central Council for Research in Homeopathy, Ministry of AYUSH, Janakpuri 110058, New Delhi, India; 7 Director General, Central Council for Research in Homeopathy, Ministry of AYUSH, Governmentof India, Janakpuri 110058, New Delhi, India

**Keywords:** immunostimulant therapy, alternative medicine, homeopathic treatment, phagocytic activity, cytokines

## Abstract

This study investigated how *Rhus toxicodendron* (RT) (6C, 30C, and 200C) can boost the immune system of BALB/c mice that were given cyclophosphamide (CPM), which is an anticancer drug that weakens the immune system. RT, known for its historical use in traditional homeopathic remedies, has demonstrated immunomodulatory and anti-inflammatory effects in various experimental models. To test the immune-boosting effects of RT, CPM (80 mg/kg) was given intraperitoneally to mice on days 4, 8, and 12 of the study but not to the normal control group. CPM-induced immunosuppression led to significant decreases in red blood cell (RBC), white blood cell (WBC), and hemoglobin (Hb) levels, and reduced spleen and thymus indices. Phagocytic activity, cytokine concentrations, and spleen architecture were also adversely affected. RT treatment, particularly at 200C, significantly ameliorated these effects, improving RBC, WBC, and Hb levels. Furthermore, RT partially prevented CPM-induced atrophy of immune organs. Treatment positively influenced cytokine production at both the protein and mRNA levels, restoring immune balance. Histopathological results confirmed that RT stimulated the immune system. The cells were more stable, and the white pulp in the spleen was arranged in a regular pattern. These findings suggest that RT may serve as an adjunctive immunostimulant therapy for conditions characterized by immunosuppression. However, further investigations in other immunocompromised states must validate these results before considering human clinical trials.

## 1. Introduction

The immune system consists of different organs, proteins, and cells that detect and respond immediately against foreign bodies by releasing different mediators and cytokines, such as interferons (IFNs) and interleukins (ILs), in a balanced state [1,2]. However, the immune system is compromised by severe infections, cancer, genetic mutations, chronic illness, etc. This compromised condition alters the equilibrium between pro- and anti-inflammatory mediators and cytokines, increasing susceptibility of the body to secondary infections [3].

The immune system is also compromised by chemotherapy for certain diseases, most commonly cancer. Furthermore, immunosuppressive medicines often increase infection susceptibility by weakening immune responses [4,5]. Therefore, investigating immunomodulatory chemicals, particularly those obtained from medicinal plants, is a promising approach for enhancing the body’s defense mechanisms via phytomedicine [6].

*Rhus toxicodendron* (RT), more widely known as poison ivy, is one of the botanical choices that can be considered [7]. Preliminary phytochemical screening findings from RT showed the existence of many types of chemicals, such as flavonoids, phenolic acids and their derivatives, catechin concoction-urushiols, and tannins. Fisetin, quercetin, kaempferol, and myricetin are examples of flavonols and their RT derivatives [8]. RT has been well-established to treat many conditions, such as skin eruptions, rheumatoid arthritis, and muscle pains, since 1798 [7,8,9,10,11,12,13], even though it produces rashes and discomfort in the musculoskeletal system due to the potent allergen urushiol. Recent investigations have consistently demonstrated the analgesic, anti-inflammatory, and immunomodulatory effects of homeopathic dilutions and crude RT in experimental animal models [7,10,13,14]. According to these investigations, including those conducted by Carvalho et al., homeopathic RT dilutions (6C, 12C, 30C, and 200C) exhibit anti-inflammatory activity by affecting the prostaglandin and histamine pathways, promoting anti-arthritis reactions [7]. However, Patil et al. observed a proinflammatory impact when the crude form of RT was given in multiple oral doses in the same model [14]. Notably, the observation that different dilutions of RT exhibit distinct effects in experimental animal models, as observed in the above-cited studies [7,10,13,14], underscores the need for a comprehensive study of the immunomodulatory properties of RT. The primary objective of the present investigation was to evaluate the immunomodulatory effects of RT in BALB/c mice with CPM-induced immunosuppression.

Cyclophosphamide (CPM) is an extensively used alkylating drug in the treatment of cancer and autoimmune disorders [15]. CPM administration can cause excessive oxidative stress due to signaling disruption as well as damage the structure of DNA and cut off its copy, resulting in cell death [16]. Furthermore, CPM can lead to an imbalance of T-helper (Th) 1 and 2 cell responses as well as a decline in the absolute number of T and B cells [17]. Long-term usage of CPM was found to have significant side effects, such as immunosuppression, myelosuppression, and leukopenia [18]. CPM-treated mice are suggested as a model for testing immunomodulation by antimicrobial drugs or immunomodulators in immunocompromised animals [15,16,17,18]. In the current investigation, CPM was used to induce immunosuppression in BALB/c mice in order to explore the influence of RT on immunological status enhancement.

## 2. Materials and Methods

### 2.1. Chemicals and Reagents

RT, at dilutions of 6C, 30C, and 200C, and dispensing alcohol (90%) were acquired from Hahnemann Publishing Co., Pvt. Ltd. (GMP certified company; ISO 9001:2008 Unit), Kolkata, India, with batch numbers of 0204 and 8345, respectively. The dilutions were made according to techniques outlined in the Homoeopathic Pharmacopoeia of India [19]. Fresh leaves of RT were macerated in 90% *v/v* alcohol to produce an ethanolic solution, known as mother tincture. Potency/potentized dilution 1C was created by diluting 1 mL of mother tincture in 99 mL of HPI-grade ethyl alcohol and succussing 10 times. Similarly, the next potencies were created by diluting 1 mL of potency 1C with 99 mL of ethyl alcohol and performing 10 jerks, and the process was repeated to obtain potentized dilutions of 6C, 30C, and 200C [20].

### 2.2. Preparation of a Stock Solution of the Drug

Stock solutions of RT-potentized dilutions and dispensing alcohol were prepared by diluting 1 mL of potentized dilution (6C, 30C, and 200C) with 9 mL of double-distilled water for administration to experimental animals [21,22], and all the solutions were blinded until the statistical analysis.

### 2.3. Experimental Animals

This study utilized 70 healthy BALB/c mice of both genders (male/female—1:1), bred and maintained in the institutional animal house of Dr. Anjali Chatterji Regional Research Institute for Homeopathy, Kolkata. The mice were maintained on a 12 h light–dark cycle, with bedding made of autoclaved corn cob (Sparconn Life Sciences Pvt. Ltd., Karnataka, India), and individually kept in a ventilated polysulfonate cage system (Citizen Industries Pvt. Ltd., Ahmedabad, India). Furthermore, the mice were fed a pelleted diet (VRK Industries Pvt. Ltd., Maharashtra, India) and water ad libitum and stored in an air-conditioned room with a relative humidity of 55–65% at 23 ± 2 °C. On average, 6–8-week-old mice weighing 18–20 g were used for the experiments. Care and experimental protocols were followed per CCSEA’s and India’s guidelines. All the protocols of Dr. Anjali Chatterjee Regional Research Institute for Homoeopathy (Reg. No. 2055/GO/RBi/S/19/CPCSEA), Kolkata (Proposal no: DACRRIH/CPCSEA/IAEC/2021/005) were approved by the IAEC. All groups were maintained under perfect hygienic conditions by adhering to the required ethical standards and following the suggested protocols.

### 2.4. Experimental Design

#### 2.4.1. Sample Size

G*Power (v.3.1) software was used to calculate the sample size for the immunomodulatory activity experiment based on priori power analysis. Ten mice per group were used for the investigation, with a statistical power of 82% and an alpha error rate of 0.05.

#### 2.4.2. Cyclophosphamide-Induced Immunosuppression Model

Mice were divided into seven groups and further segregated into two subgroups of five males and five females each. The normal control group was designated Group-I, and it received distilled water (40 µL/100 g, p.o.). Group-II received distilled water (40 μL/100 g, p.o.) and CPM (80 mg/kg) and served as the disease control. Group-III received dispensing alcohol (40 μL/100 g, p.o.) and served as the vehicle control. Group IV animals were treated with levamisole (40 mg/kg, p.o.); groups V, VI, and VII received RT orally (40 μL/100 g, p.o.) in 6C, 30C, and 200C potencies for 14 days, respectively. On the 4th, 8th, and 12th days of treatment, all groups received 80 mg/kg of CPM intraperitoneally (IP) to induce immunosuppression [23], except for the normal control group.

Blood was collected from the animals by puncturing the retro-orbital plexus 24 h after the last dose was administered while the animals were under isoflurane anesthesia (Raman and Weil Pvt. Ltd., Daman, India) using a small-animal anesthesia system (Orchid Scientific Pvt. Ltd., Nashik, India). The collected blood was stored in vials containing the necessary amount of anticoagulant. A veterinary hematology analyzer (Genvet-VH 50, Genrui Biotech Inc., Shenzhen, China) was utilized to determine the counts of red blood cells (RBCs), white blood cells (WBCs), hemoglobin (Hb), and platelets.

### 2.5. Calculation of Immune Organ Indices

Twenty-four hours after the last dose was administered, the mice were sacrificed via cervical dislocation, and the thymus and spleen were removed and weighed aseptically. The immune organ indices were calculated as follows [23]:

(1) Immune Organ Index (%) (Spleen or Thymus)=(Spleen or Thymus weight)/(Body Weight)×100

### 2.6. Phagocytic Index Assay

A carbon clearance test was performed to evaluate the efficacy of the test drugs on phagocytic activity of the reticuloendothelial system. The test was conducted to determine the phagocytic index using the methodology outlined by Cheng et al. in 2005 and Raj and Gothandam in 2015 [24,25] with slight modifications. On the 14th day of the experiment, the mice (*n* = 4) were weighed and then injected with prewarmed Indian ink (0.1 mL/10 g body weight) via the tail vein 2 h after the last dose was administered. Blood samples were obtained 2 and 10 min after injection via retro-orbital puncture. A 25 μL blood sample was combined with 2 mL of a 0.1% sodium carbonate (Na_2_CO_3_) solution. The absorbance of the mixture was then measured at a wavelength of 660 nm, using the Na_2_CO_3_ solution as a reference. Subsequently, the mice were euthanized using cervical dislocation, and the spleen and liver were removed and promptly weighed. The carbon clearance rate (κ) and phagocytic index (α) were determined using the following calculations:

(2) Carbon clearance rate (κ)=(logOD1−logOD2)/T2−T1

(3) Rate of phagocytic index (α)=3√κ×Body Weight/(Liver Weight+Spleen Weight) 

### 2.7. Determination of Cytokines in Serum and Spleen by ELISA

Mouse-specific ELISA kits (ABclonal, Woburn USA) were used to determine cytokine levels in the serum and spleen of the test animals (*n* = 6). Similarly, the manufacturer’s protocol was strictly followed. Serum was obtained from the blood collected. For the other part of the experiment, the protocol described by Han et al. (2019) with minor modifications was followed [26]. After perfusion, part of the spleen was weighed accurately and homogenized in an extraction buffer, and the temperature was maintained at 4 °C throughout the process. The homogenate was cold-centrifuged at 3500 rpm for 15–20 min. The supernatant was removed by pipetting, and further analysis was performed using an ELISA reader (Bio-Rad, Hercules, CA, USA) following the manufacturer’s protocol.

### 2.8. Gene Expression Analysis

Twenty-four hours after the last dose, the mice (*n* = 6) were sacrificed, and spleens were collected in sterile microcentrifuge tubes (previously autoclaved). Spleens were weighed, collected in a prechilled microcentrifuge tube, and placed on an ice box to prevent RNA degradation. The spleen was stored at −80 °C for further processing. A spleen tissue of ~100 mg was used for RNA extraction. An Aurum Total RNA Mini Kit (Bio-Rad, USA) was used to isolate the RNA successfully according to the manufacturer’s instructions. RNA purity was checked and quantified using a NanoDrop™ One Microvolume UV-Vis spectrophotometer. An iScript cDNA Synthesis Kit was used to synthesize cDNA from mRNA according to the manufacturer’s guidelines. cDNA was further diluted 10 times and stored for qRT-PCR. The mRNA expression levels of cytokines IL-2, IL-4, TNF-ɑ, IFN-γ, and glyceraldehyde-3-phosphate dehydrogenase (GAPDH)] were determined. GAPDH, which is a housekeeping gene, is an internal control for cytokine expression that accounts for variations in mRNA loading. The primers used for cytokines IL-2, IL-4, TNF-ɑ, IFN-γ, and GAPDH were selected from Table 1. An iTaq Universal SYBR Green Supermix and CFX96 Real-Time Fluorescence Quantitative PCR Detection Equipment from Bio-Rad (USA) were used for real-time PCR analysis. The relative quantification of objective genes was calculated according to the 2^−ΔΔCt^ method [27].

### 2.9. Histopathological Analysis (H&E Staining) of the Spleen

After the whole spleen was collected, it was washed in sterile phosphate-buffered saline (PBS) (pH 7.3 ± 0.1) and kept in a buffered formalin solution for 24 h. After the specified duration, the tissue was trimmed into 3–5 mm thick pieces for final sectioning. Once the tissue was fixed correctly, it was incubated in stronger alcohol solutions. Once completely dry, the tissue was incubated in xylene before being embedded in liquid paraffin to make paraffin-embedded tissue blocks. Tissue sections 3–5 µm thick from the specimen slides were prepared using a semiautomated microtome (Biocraft CIM 18, Agra, India). These slides were stained with hematoxylin and eosin (HE) to analyze the splenic histoarchitecture [23].

### 2.10. Statistical Analysis

The data are presented as the mean ± standard error of the mean (SEM). One-way ANOVA and Tukey’s multiple comparison tests were used to compare various groups. The statistical analyses were performed using SPSS 26 software. Statistical significance was determined based on *p* values less than 0.05, 0.01, and 0.001.

## 3. Results

### 3.1. Effect of RT on RBC, WBC, and Hb in CPM-Treated Mice

**Figure 1:**
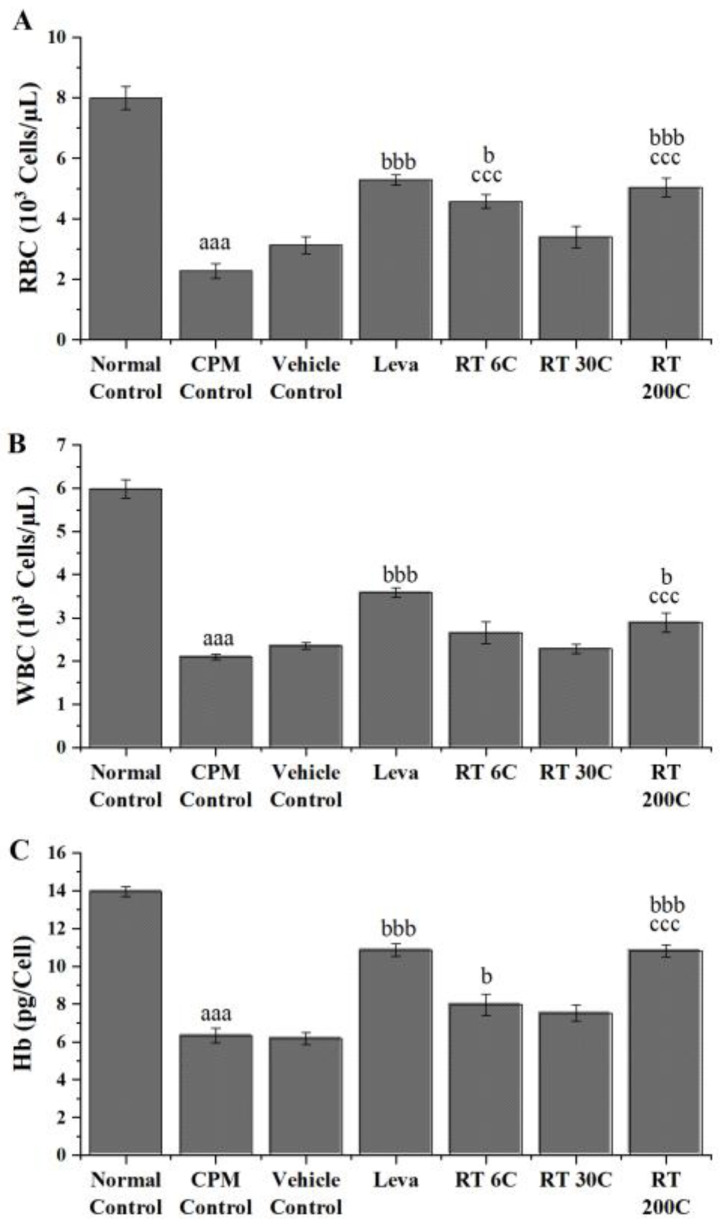
Effect of RT on hematological parameters in CPM-treated mice; (**A**) RBC count, (**B**) WBC count, and (**C**) hemoglobin. The data are presented as the mean ± SEM (*n* = 6). The data were analyzed using one-way ANOVA followed by Tukey’s multiple comparison post hoc test. ^aaa^ *p* < 0.001 compared to the normal control group; ^b^ *p* < 0.05, and ^bbb^ *p* < 0.001 compared to the CPM group; ^ccc^ *p* < 0.001 compared to the vehicle control group.

RBC, WBC, and Hb counts were assessed in animals treated with CPM and found to be significantly lower (*p* < 0.001) than those in the normal control group, indicating the induction of immunosuppression by CPM. In immunosuppressed mice, preventive treatments with RT at 6C and 200C significantly increased RBC levels (*p* < 0.01 and *p* < 0.001, respectively). In the case of WBCs, RT at 200C showed a substantial increase (*p* < 0.05), and at 6C and 200C significantly increased Hb levels (*p* < 0.05 and *p* < 0.001) in CPM-treated mice. In contrast to the vehicle control, RT significantly improved RBC, WBC, and Hb levels, indicating that the activity observed was due to the presence of the drug but not the vehicle used, i.e., alcohol. Compared with the CPM control, levamisole treatment also significantly improved RBC, WBC, and Hb levels (*p* < 0.001). The results indicated that RT could reverse CPM-induced suppression of RBC, WBC, and Hb in mice, and 200C RT was more effective than 6C and 30C RT (Figure 1).

### 3.2. Effect of RT on Thymus and Spleen Indices in the CPM-Treated Group

**Figure 2:**
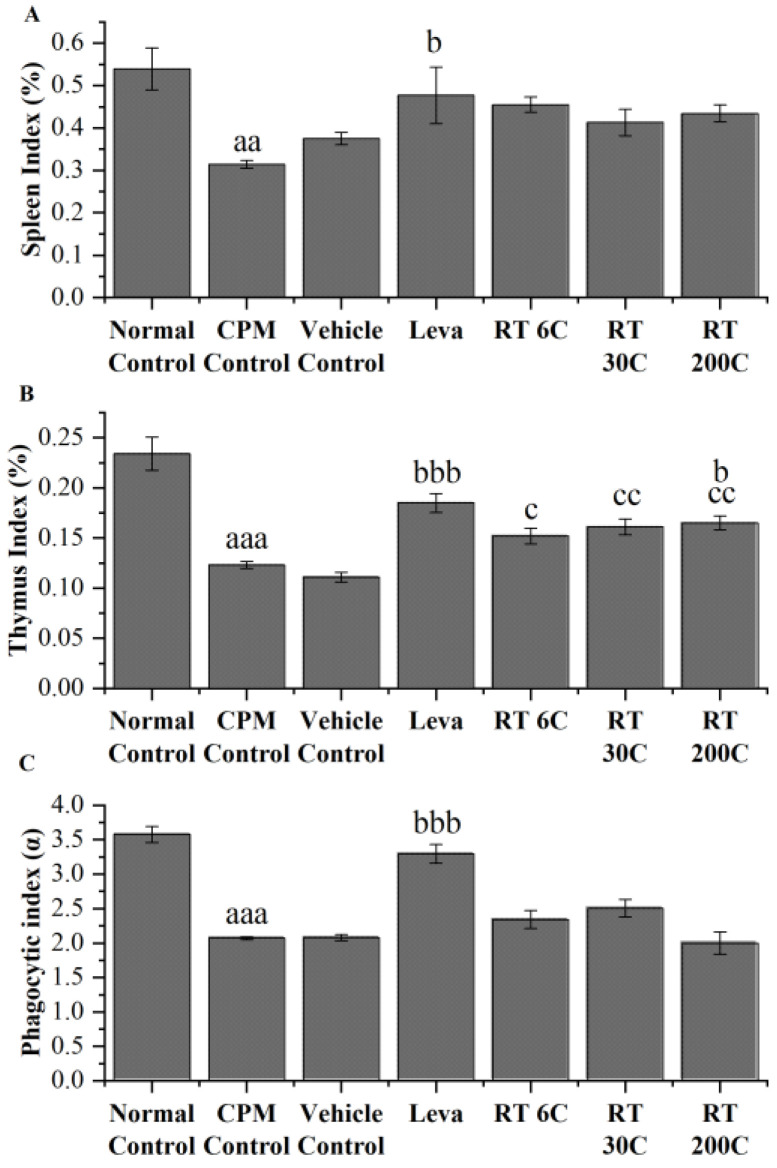
Effect of RT on immune indices in CPM-treated mice; (**A**) spleen index (*n* = 6), (**B**) thymus index (*n* = 6), and (**C**) phagocytic index (*n* = 4). The data are presented as the mean ± SEM. The data were analyzed using one-way ANOVA followed by Tukey’s multiple comparison post hoc test. ^aa^ *p* < 0.01 and ^aaa^ *p* < 0.001 compared to the normal control group; ^b^ *p* < 0.05, and ^bbb^ *p* < 0.001 compared to the CPM group; ^c^ *p* < 0.05 and ^cc^ *p* < 0.01 compared to the vehicle control group.

In mice treated with CPM, spleen and thymus indices were significantly lower (*p* < 0.01 and *p* < 0.001, respectively) than those in the normal control group. The 6C, 30C, and 200C potencies of RT also did not significantly improve the spleen index in immunocompromised BALB/c mice. The 200C potency alone significantly improved (*p* < 0.05) the thymus index, and 6C and 30C also improved the thymus index, but the differences were not statistically significant. Compared with vehicle, RT significantly improved the thymus index, indicating that the effect was due to RT but not to the vehicle. Levamisole-treated mice exhibited significant improvements in spleen (*p* < 0.05) and thymus (*p* < 0.001) indices. The above data suggested that RT could partially prevent the reduction in CPM-induced decreases in spleen and thymus indices (Figure 2A,B).

### 3.3. Effect of RT on the Phagocytic Index in CPM-Treated Mice

Similar to the immune organ indices, the phagocytic index of CPM-treated animals was markedly lower (*p* < 0.001) than that of the normal control animals. The administration of RT (6C, 30C, and 200C) slightly enhanced phagocytic function. However, this improvement did not reach statistical significance compared to that of the mice in the CPM group. The phagocytic indices of CPM-treated levamisole-treated mice were significantly greater than those of normal control mice (Figure 2C).

### 3.4. Effect of RT on Cytokine Levels in the Serum and Spleen of CPM-Treated Mice

**Figure 3:**
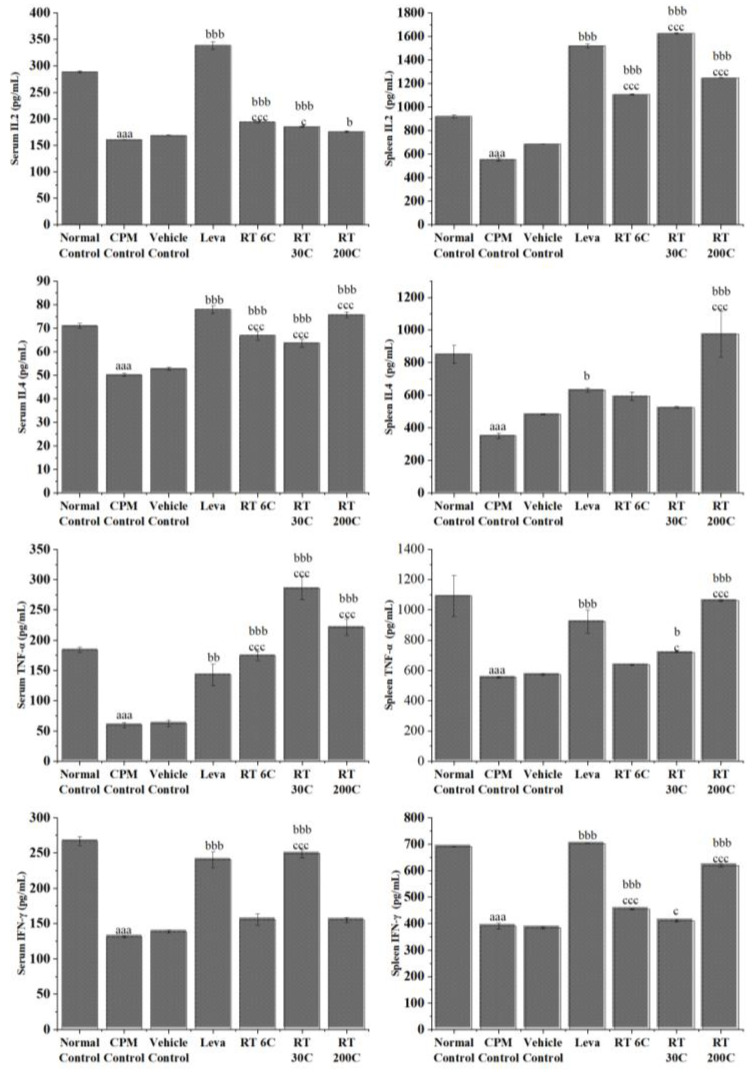
Effect of RT on serum and spleen cytokine levels in CPM-treated mice. The data are presented as means ± SEM (*n* = 6). The data were analyzed using one-way ANOVA followed by Tukey’s multiple comparison post hoc test. ^aaa^ *p* < 0.001 compared to the normal control group; ^b^ *p* < 0.05, ^bb^ *p* < 0.01, and ^bbb^ *p* < 0.001 in contrast to the CPM group; ^c^ *p* < 0.05, and ^ccc^ *p* < 0.001 in comparison with the vehicle control group.

CPM treatment caused a significant reduction in the levels of cytokines (IL-2, IL-4, TNF-α, and IFN-γ) (*p* < 0.001) in the serum and splenic tissue of mice, indicating its immunosuppressive effect. The serum and splenic levels of IL-2 were significantly greater in the Rhus-toxin-treated groups (6C, 30C, and 200C) than in the CPM control group (*p* < 0.05/*p* < 0.01/*p* < 0.001). RT significantly increased serum TNF-α concentration at 6C, 30C, and 200C. In contrast, only 30C- and 200C-potency-treated animals exhibited increased (*p* < 0.05 and *p* < 0.001, respectively) splenic TNF-α levels compared to those in the CPM control group. Treatment with RT at 6C, 30C, and 200C significantly increased (*p* < 0.001) serum IL-4 concentration, whereas treatment with 200C alone significantly increased splenic IL-4 concentration compared to that in the CPM control group. Splenic levels of IFN-γ were considerably increased by RT at 6C and 200C, whereas only 30C-potency-treated animals exhibited significantly improved (*p* < 0.001) serum IFN-γ levels compared to those in the CPM control group. The positive control (levamisole) significantly enhanced the secretion of IL-2, IL-4, TNF-α, and IFN-γ in both the serum and spleen compared with that in the CPM-treated group (*p* < 0.05/*p* < 0.01/*p* < 0.001). These results indicate that RT could enhance immune activity by improving cytokine production in the serum and splenocytes of immunosuppressed mice. Figure 3 shows the serum and splenic levels of these cytokines in mice treated with RT.

### 3.5. Effect of RT on the mRNA Expression Levels of Cytokines (IL2, IL4, TNF-α, and IFN-γ) in the Spleens of CPM-Treated Mice

**Figure 4:**
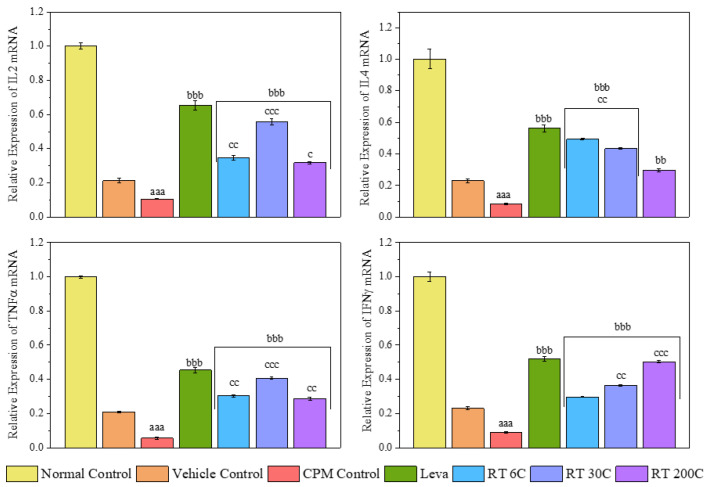
Effect of RT on the mRNA expression levels of cytokines in the spleens of CPM-treated mice. The data are presented as means ± SEM (*n* = 6). The data were analyzed using one-way ANOVA followed by Tukey’s multiple comparison post hoc test. ^aaa^ *p* < 0.001 compared to the normal control group; ^bb^ *p* < 0.01 and ^bbb^ *p* < 0.001 in contrast to the CPM group; ^c^ *p* < 0.05, ^cc^ *p* < 0.01, and ^ccc^ *p* < 0.001 in comparison with the vehicle control group.

To further confirm the splenic immune enhancement mediated by RT, qRT-PCR was conducted to examine the induction of cytokine transcription in the spleen. As shown in Figure 4, the 6C, 30C, and 200C potencies substantially increased (*p* < 0.01/*p* < 0.001) the mRNA levels of IL-2, IL-4, TNF-α, and IFN-γ, while CPM-treated mice exhibited significantly decreased (*p* < 0.001) mRNA levels of these cytokines. Compared with those in CPM-treated mice, levamisole treatment also significantly upregulated (*p* < 0.001) the mRNA expression of genes encoding IL2, IL4, TNF-α, and IFN-γ. The mRNA expression of the above cytokines significantly differed between the RT- and vehicle-treated groups, further confirming that RT was responsible for reversing the downregulation of the mRNA expression of cytokines induced by CPM in mice.

### 3.6. Effect of RT on Spleen Histology in CPM-Treated Mice

**Figure 5:**
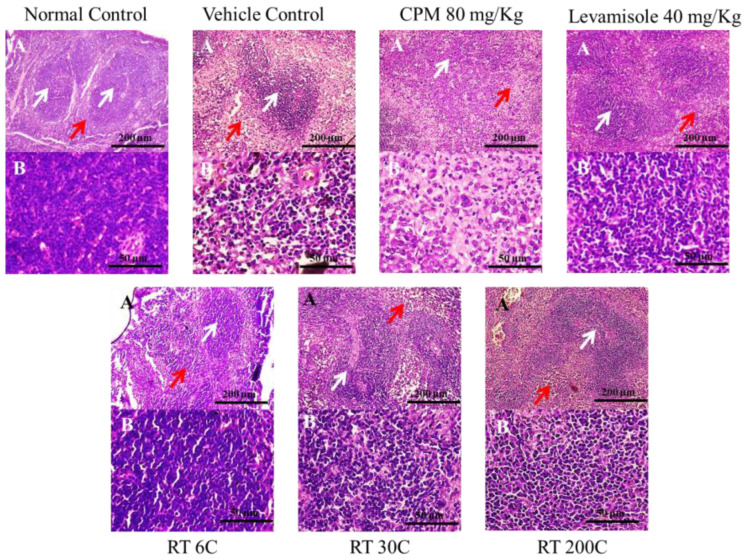
Effect of RT on the histology of HE-stained spleen sections from CPM-treated mice; plate (A), with a 10× objective focused on both white and red pulp; plate (**B**), with a 40× objective focused on white pulp containing lymphocytes; the red arrow and white arrow indicate red pulp and white pulp, respectively.

A histological examination of HE-stained spleen sections from normal control mice revealed a healthy, well-defined architecture of splenic tissue with a regular arrangement of white and red pulp, as shown in Figure 5. The nuclei were visible with distinct boundaries. However, treatment with CPM at a dose of 80 mg/kg body weight resulted in an irregular arrangement of follicles with diffuse and distorted white pulp. The white pulp contained fewer lymphocytes, and an increase in apoptotic cells was observed in the spleen.

When immunocompromised mice were subjected to alcohol treatment, i.e., the vehicle control group, white pulp underwent degeneration and distortion, causing lymphocytes to migrate toward red pulp. Additionally, fibrosis was observed in the spleen. In contrast, treatment with RT at 6C, 30C, and 200C markedly improved the histoarchitecture of the spleen. Red and white pulp were appropriately arranged, and there was an increase in the lymphocyte count. The necrotic regions were less prominent than those in the control group. The same positive changes were observed with levamisole treatment.

## 4. Discussion

Immunosuppression, characterized by a temporary or permanent deficiency in immunity, can render the body susceptible to external pathogens [28]. In the realm of homoeopathy, where the focus lies on stimulating the body’s inherent healing mechanisms, the exploration of immune-enhancing agents from medicinal plant origins aligns with holistic healing principles [29]. Homoeopathic principles underscore treating the whole person rather than merely the symptoms, aiming to strengthen the vital force and enhance the body’s ability to defend itself [30]. Therefore, the pursuit of immune-enhancing agents within the realm of homoeopathy reflects a dedicated effort to harness the healing potential of nature and address immunosuppression through a holistic and individualized approach to healthcare. Thus, in the present study, the immunomodulatory potential of ultra-high dilutions of RT was comprehensively investigated in BALB/c mice subjected to CPM-induced immunosuppression.

CPM (80 mg/kg) was intraperitoneally injected into mice in all groups, except the normal control group, to induce immunosuppression. By comparing the CPM group and control group, we observed that CPM significantly reduced RBC, WBC, and Hb levels, and decreased spleen and thymus indices. CPM also decreased the phagocytic index and serum and splenic IL2, IL4, TNF-α, and IFN-γ concentrations. Moreover, splenic mRNA expression of IL2, IL4, TNF-α, and IFN-γ in CPM mice decreased significantly. These results are reflected considerably in the histology and result in an irregular arrangement of follicles with diffuse and distorted white pulp, suggesting that CPM could disrupt the normal architecture of the spleen. The data above indicate a successful mouse model using CPM that effectively suppressed the immune system, which aligns with earlier findings [31,32,33].

Treatment with RT (6C and 200C) significantly reversed the decrease in RBC and Hb levels induced by CPM in mice. RT at 200C also significantly improved the WBC count in CPM-treated mice. The results indicate that RT treatment could enhance immunity in mice by improving RBC, WBC, and Hb levels reduced by CPM. At the same time, the body’s innate immune system is reflected in the weight of critical immunological organs, i.e., the spleen and thymus [34]. The efficiency of the innate immune system is reflected in alterations observed in the indices of the thymus and spleen, where the thymus serves as the site for immunological cell development and maturation. In contrast, the spleen functions as a peripheral organ where fully matured immune cells reside and actively participate in responding to pathogen invasion or disease conditions [35]. Under pathological conditions, shrinkage of the spleen and thymus and a decrease in their weight represent a decrease in immune function [36]. The RT treatment in this trial resulted in a partial improvement in spleen and thymus indices compared to those in the CPM group, but the differences were not significant. This suggests that RT may protect against the shrinkage of immunological organs caused by CPM. Furthermore, the phagocytic activity of RT was investigated through a carbon clearance test, and it was found that the phagocytic activity of macrophages was reduced by CPM administration, which concurs with earlier studies [37,38]. Compared with the CPM control, RT treatment improved the phagocytic activity of macrophages, but the results were not statistically significant.

Given that cytokines play a vital role in the development of an immune response, the effect of RT on the production of IL-2, IL-4, TNF-α, and IFN-γ was evaluated. A large body of cellular and animal study data also showed that promoting cytokine synthesis improved immune function in immunocompromised mice [33,37,39,40,41]. Cytokines serve as signal transduction mediators between cells, playing a role in inflammatory reactions, immune cell differentiation, and the stimulation of hematopoietic functions [42]. IL-2 is a cytokine that stimulates cell development and facilitates cell division and differentiation. IL-2 can promote the synthesis of interferon and plays a role in inflammatory reactions. TNF-α is essential for the body’s defense mechanism and kills tumor cells. TNF-α stimulates the expression of interleukins (IL-1, IL-6, and IL-8), chemokines (RANTES and MCP-1), adhesion molecules (VCAM-1 and ICAM-1), proinflammatory proteins, acute phase proteins (CRP and SAA), and matrix metalloproteinases, which play pivotal roles in immune and inflammatory responses [43]. IFN-γ exerts its biological effects by inhibiting viral replication, activating macrophages, and inducing the production of major histocompatibility (MHC) molecules. IL-4 is secreted by Th2 cells, mast cells, and natural killer (NK) cells to act upon T cells, B cells, macrophages, and mast cells, resulting in the activation and differentiation of B cells, the upregulation of class II MHC expression, and the induction and growth of T cells and mast cells, respectively [25,32,44]. Our analyses showed that RT had an excellent regulatory effect on cytokine production in CPM-induced immunosuppressed mice. At 6C, 30C, and 200C potencies, RT increased concentrations of IL-2, TNF-α, IFN-γ, and IL-4 in mouse serum. Moreover, the same trend was observed for splenic cytokines, indicating the effect of RT on splenocytes. This outcome demonstrated that RT can modify immune system imbalance and enhance symptoms of immunosuppression.

To further confirm the immune enhancement of RT in the spleen from a CPM-induced immunosuppressed state, qRT-PCR was performed on spleen cytokines, i.e., IL-2, IL-4, TNF-α, and IFN-γ. The findings demonstrated a notable increase in the mRNA levels of IL-2, IL-4, TNF-α, and IFN-γ in the spleens of mice treated with RT. In contrast, the CPM group had reduced mRNA levels of these cytokines. The current study revealed that RT led to a notable increase in the mRNA expression of cytokines, suggesting that it may have immunostimulant effects in mice with CPM-induced immunosuppression.

Histopathological findings provided more evidence for the immunostimulant effects of RT, as demonstrated by its ability to protect the spleen. Compared to the CPM control, the administration of RT resulted in significant enhancements in spleen histoarchitecture. Histopathological examinations revealed that RT treatment increased the cellular integrity and organized structure of white pulp in the spleen, indicating that the treatment contributed to enhancing the immune system and supporting proper lymphocyte function.

These results also suggest that the presence of alcohol in RT as vehicle does not have a noticeable impact on the immunostimulant effects of RT. This was evidenced by the absence of progress in the CPM-treated mice that were given alcohol only. In addition, the immunoenhancement activity of RT (6C, 30C, and 200C) was close to levamisole.

Several reports suggest that ultra-high dilutions of homeopathic medicines may exert their therapeutic effects through their impact on gene expression [45,46,47,48,49,50,51]. In our current study, we observed non-linear effects on the serum and splenic levels of cytokines, as well as their mRNA expression in the spleen when treated with high RT dilutions (6C, 30C, and 200C). These cytokines might have initiated endogenous cellular events, leading to cell-to-cell signaling through mediators to execute the observed effects [52]. These findings prove the immunostimulatory properties of highly diluted RT in CPM-induced immunosuppression.

## 5. Conclusions

To summarize, this work demonstrated the ability of diluted RT to modulate the immune system in BALB/c mice immunosuppressed by CPM. RT treatment alleviated the decreases in important blood parameters, immune organ sizes, and cytokine levels caused by CPM, demonstrating its ability to combat immunosuppression. This study confirmed that RT has a protective effect on the structure of the spleen and indicated its ability to strengthen the immune system. These findings emphasize the potential of RT as an adjuvant agent for combating immunosuppression. Further research is needed to explore its effectiveness in a broader range of immunocompromised states before considering clinical trials in humans.

## Figures and Tables

**Table 1:** Primer sequences for RT-PCR.

Gene	Sequence 5′-3′	Size bp
IL-2	F-GCAGCTGTTGATGGACCTAC	20
	R-TCCACCACAGTTGCTGACTC	20
IL-4	F-TCGGCATTTTGAACGAGGTC	20
	R-GAAAAGCCCGAAAGAGTCTC	20
TNF-α	F-ATGAGCACAGAAAGCATGATC	21
	R-TACAGGCTTGTCACTGGAATT	21
IFN-γ	F-TGAGCAGAGCTCTTGTGGTC	20
	R-CGTTCCTCCTTGTGGCCTAA	20
GAPDH	F-5′GTGGAGTCTACTGGTGTCTTC′3	21
	R-5′GTGCAGGAGGCATTGCTTACA3′	21

## Data Availability

The study material is stored in the archive of the test facility of Dr. Anjali Chatterji Regional Research Institute for Homeopathy. The study material includes electronic backup and print copies of the study plan, raw data, Microsoft Excel-compiled raw data, statistical output data, and the final report. The materials will be retained for at least 5 years from archiving of the final report. After that, as per the directions of the competent authority, the archived materials may be disposed of, or the archival period may be extended.

## References

[1] Loh L., Wang Z., Sant S., Koutsakos M., Jegaskanda S., Corbett A.J., Liu L., Fairlie D.P., Crowe J., Rossjohn J. (2016). Human mucosal-associated invariant T cells contribute to antiviral influenza immunity via IL-18–dependent activation. Proc. Natl. Acad. Sci. USA.

[2] Ferlazzo G., Tsang M.L., Moretta L., Melioli G., Steinman R.M., Munz C. (2002). Human dendritic cells activate resting natural killer (NK) cells and are recognized via the NKp30 receptor by activated NK cells. J. Exp. Med..

[3] Shruthi S., Vijayalaxmi K., Shenoy K.B. (2018). Immunomodulatory effects of gallic acid against cyclophosphamide-and cisplatin-induced immunosuppression in Swiss albino mice. Indian J. Pharm. Sci..

[4] Wang G., Zhang Y., Zhou X., Yang M., Ma X., Liu X. (2022). Norcantharidin alleviates cyclophosphamide-induced immunosuppression via circBCL2L1/miR-30c-3-3p/TRAF6 axis. Qual. Assur. Saf. Crops Foods.

[5] Winkelstein A. (1973). Mechanisms of immunosuppression: Effects of cyclophosphamide on cellular immunity. Blood.

[6] Hussain K., Iqbal Z., Zahid Abbas R., Kasib Khan M., Kashif Saleemi M. (2017). Immunomodulatory activity of Glycyrrhiza glabra extract against mixed Eimeria infection in chickens. Int. J. Agric. Biol..

[7] Dos Santos A., Perazzo F., Cardoso L., Carvalho J. (2007). In vivo study of the anti-inflammatory effect of Rhus toxicodendron. Homeopathy.

[8] Goswami A., Saka V.P., GV N.K., Biswas B. (2022). A Comprehensive Review on Phytochemistry and Pharmacology of Homoeopathic Medicine Rhus Toxicodendron. Homœopathic Links.

[9] Pfaff F. (1897). On the active principle of Rhus toxicodendron and Rhus venenata. J. Exp. Med..

[10] Patil C., Salunkhe P., Gaushal M., Gadekar A., Agrawal A., Surana S. (2009). Immunomodulatory activity of Toxicodendron pubescens in experimental models. Homeopathy.

[11] Xia Z., Miyakoshi T., Yoshida T. (2004). Lipoxygenase-catalyzed polymerization of phenolic lipids suggests a new mechanism for allergic contact dermatitis induced by urushiol and its analogs. Biochem. Biophys. Res. Commun..

[12] Vickers A., Zollman C. (2001). ABC of Complementary Medicine.

[13] Patil C.R., Rambhade A.D., Jadhav R.B., Patil K.R., Dubey V.K., Sonara B.M., Toshniwal S.S. (2011). Modulation of arthritis in rats by Toxicodendron pubescens and its homeopathic dilutions. Homeopathy.

[14] Patil C.R., Gadekar A.R., Patel P.N., Rambhade A., Surana S.J., Gaushal M.H. (2009). Dual effect of Toxicodendron pubescens on Carrageenan induced paw edema in rats. Homeopathy.

[15] Alam M.F., Ajeibi A.O., Safhi M.H., Alabdly A.J.A., Alshahrani S., Rashid H., Qadri M., Jali A.M., Alqahtani S., Nomier Y. (2023). Therapeutic Potential of Capsaicin against Cyclophosphamide-Induced Liver Damage. J. Clin. Med..

[16] Yadav V., Krishnan A., Zahiruddin S., Ahmad S., Vohora D. (2023). Amelioration of Cyclophosphamide-Induced DNA Damage, Oxidative Stress, and Hepato- and Neurotoxicity by Piper Longum Extract in Rats: The Role of ΓH2AX and 8-OHdG. Front. Pharmacol..

[17] Sato-Okabayashi Y., Isoda K., Heissig B., Kadoguchi T., Akita K., Kitamura K., Shimada K., Hattori K., Daida H. (2020). Low-dose oral cyclophosphamide therapy reduces atherosclerosis progression by decreasing inflammatory cells in a murine model of atherosclerosis. IJC Hear. Vasc..

[18] Quan X., Chen H., Liang S., Yang C., Yao C., Xu Y., Liu H., An N. (2022). Revisited Cyclophosphamide in the Treatment of Lupus Nephritis. Biomed Res. Int..

[19] Ministry of Health & Family Welfare(India) (1990). Homoeopathic Pharmacopoeia of India.

[20] Goswami A., Kumar G.V.N., Saka V.P., Gupta P., Verma D. (2024). Safety Assessment of Ultra High Dilutions of Camphora Officinarum: A Preclinical Investigation. Altern. Ther. Health Med..

[21] Lal R., Sharma M., Behera S., Regar R.K., Tripathi D., Kumar G.N., Singh S., Verma D., Gupta P., Kaushik S. (2023). Safety Evaluation of Arsenicum Album in Acute and Sub-Acute Toxicity Studies in Rats. Toxicol. Int..

[22] Saka V.P., Kumar G.N., Goswami A., Verma D., Gupta P. (2023). Camphora Augments Humoral Mediated Immunity and Decreases Delayed type Hypersensitivity in BALB/c Mice. J. Nat. Remedies.

[23] Yan H., Lu J., Wang J., Chen L., Wang Y., Li L., Miao L., Zhang H. (2021). Prevention of cyclophosphamide-induced immunosuppression in mice with traditional Chinese medicine Xuanfei Baidu decoction. Front. Pharmacol..

[24] Cheng W., Li J., You T., Hu C. (2005). Anti-inflammatory and immunomodulatory activities of the extracts from the inflorescence of Chrysanthemum indicum Linne. J. Ethnopharmacol..

[25] Raj S., Gothandam K. (2015). Immunomodulatory activity of methanolic extract of Amorphophallus commutatus var. wayanadensis under normal and cyclophosphamide induced immunosuppressive conditions in mice models. Food Chem. Toxicol..

[26] Han L., Meng M., Guo M., Cheng D., Shi L., Wang X., Wang C. (2019). Immunomodulatory Activity of a Water-Soluble Polysaccharide Obtained from Highland Barley on Immunosuppressive Mice Models. Food Funct..

[27] Livak K.J., Schmittgen T.D. (2001). Analysis of relative gene expression data using real-time quantitative PCR and the 2^−ΔΔCT^ method. Methods.

[28] Kim H.-R., Kim Y.-S., Lee D.-R., Choi B.-K., Kwon K.-B., Bae G.-S. (2021). Echinacea purpurea alleviates cyclophosphamide-induced immunosuppression in mice. Appl. Sci..

[29] House of Commons Science and Technology Committee (2010). Evidence Check 2: Homeopathy. https://www.google.com.hk/url?sa=t&source=web&rct=j&opi=89978449&url=https://publications.parliament.uk/pa/cm200910/cmselect/cmsctech/45/45.pdf&ved=2ahUKEwjt5azRhryHAxX7sVYBHe5IBE4QFnoECBkQAQ&usg=AOvVaw3ZMuHyefSTMUsVoOYb79I5.

[30] Vithoulkas G. (2017). Serious mistakes in meta-analysis of homeopathic research. J. Med. Life.

[31] Zhou X., Dong Q., Kan X., Peng L., Xu X., Fang Y., Yang J. (2018). Immunomodulatory activity of a novel polysaccharide from Lonicera japonica in immunosuppressed mice induced by cyclophosphamide. PLoS ONE.

[32] Zhang W.-N., Gong L.-L., Liu Y., Zhou Z.-B., Wan C.-X., Xu J.-J., Wu Q.-X., Chen L., Lu Y.-M., Chen Y. (2020). Immunoenhancement effect of crude polysaccharides of Helvella leucopus on cyclophosphamide-induced immunosuppressive mice. J. Funct. Foods.

[33] Yingjian L., Junming H., Min C., Chenyue L., Dachao Z., Yuanhua H., Zhi L. (2013). A health food high-peptide meal alleviates immunosuppression induced by hydrocortisone and cyclophosphamide in mice. Food Funct..

[34] Barrea L., Di Somma C., Muscogiuri G., Tarantino G., Tenore G.C., Orio F., Colao A., Savastano S. (2018). Nutrition, inflammation and liver-spleen axis. Crit. Rev. Food Sci. Nutr..

[35] Yan C., Qu H., Li X., Feng B. (2023). Holothurian Wall Hydrolysate Ameliorates Cyclophosphamide-Induced Immunocompromised Mice via Regulating Immune Response and Improving Gut Microbiota. Int. J. Mol. Sci..

[36] Baqai F.P., Gridley D.S., Slater J.M., Luo-Owen X., Stodieck L.S., Ferguson V., Chapes S.K., Pecaut M.J. (2009). Effects of spaceflight on innate immune function and antioxidant gene expression. J. Appl. Physiol..

[37] Mei Y.-X., Chen H.-X., Zhang J., Zhang X.-D., Liang Y.-X. (2013). Protective effect of chitooligosaccharides against cyclophosphamide-induced immunosuppression in mice. Int. J. Biol. Macromol..

[38] Hou H., Fan Y., Wang S., Si L., Li B. (2016). Immunomodulatory activity of Alaska pollock hydrolysates obtained by glutamic acid biosensor–Artificial neural network and the identification of its active central fragment. J. Funct. Foods.

[39] Woo S.M., Choi W.R., Jang D., Yi C.S., Kim H.L., Kim K.H., Kim J.T., Choi W.H., Jang S.H., Kim M.J. (2018). Immune enhancement effect of an herb complex extract through the activation of natural killer cells and the regulation of cytokine levels in a cyclophosphamide-induced immunosuppression rat model. Asian Pac. J. Trop. Med..

[40] Kumar V.P., Venkatesh Y.P. (2016). Alleviation of cyclophosphamide-induced immunosuppression in Wistar rats by onion lectin (Allium cepa agglutinin). J. Ethnopharmacol..

[41] Gao S., Hong H., Zhang C., Wang K., Zhang B., Han Q.-A., Liu H., Luo Y. (2019). Immunomodulatory effects of collagen hydrolysates from yak (Bos grunniens) bone on cyclophosphamide-induced immunosuppression in BALB/c mice. J. Funct. Foods.

[42] Dirchwolf M., Podhorzer A., Marino M., Shulman C., Cartier M., Zunino M., Paz S., Muñoz A., Bocassi A., Gimenez J. (2016). Immune dysfunction in cirrhosis: Distinct cytokines phenotypes according to cirrhosis severity. Cytokine.

[43] Sprague A.H., Khalil R.A. (2009). Inflammatory cytokines in vascular dysfunction and vascular disease. Biochem. Pharmacol..

[44] Meng Y., Li B., Jin D., Zhan M., Lu J., Huo G. (2018). Immunomodulatory activity of Lactobacillus plantarum KLDS1. 0318 in cyclophosphamide-treated mice. Food Nutr. Res..

[45] Saha S.K., Roy S., Khuda-Bukhsh A.R. (2015). Ultra-Highly Diluted Plant Extracts of Hydrastis Canadensis and Marsdenia Condurango Induce Epigenetic Modifications and Alter Gene Expression Profiles in HeLa Cells in Vitro. J. Integr. Med..

[46] Mohan T., Periandavan K., Nayak D. (2022). Evaluation of Hypolipidemic Activity of Homeopathic Drug Allium Sativum 6C Potency on Different Grades of Dyslipidemia in Wistar Albino Rat Models. Phytomed. Plus.

[47] Joshi J., Bandral C., Manchanda R.K., Khurana A., Nayak D., Kaur S. (2022). Evidence for Reversal of Immunosuppression by Homeopathic Medicine to a Predominant Th1-Type Immune Response in BALB/c Mice Infected with Leishmania Donovani. Homeopathy.

[48] Saxena S.K., Kumar S., Maurya V.K., Nayak D., Kaushik S., Manchanda R.K., Gadugu S. (2023). Antiviral and Anti-Inflammatory Activity of Novel Belladonna Formulation against Japanese Encephalitis Virus via Inhibition of P65 Nuclear Translocation and TNF-α Mediated NF-KB Signaling. Biotechnology and Genetic Engineering Reviews.

[49] Sarkar A., Roy A., Maity M., Nayak D., Das S. (2023). Ultradiluted Eupatorium Perfoliatum Alleviates DENV-Induced Fibrosis By Regulation of TGFβ1, MMP-9 and Interferons. Future Virol..

[50] Mohan T., Rajkumar A., Panchalingam G., Nayak D., Raghunathan M., Periandavan K. (2024). Homeopathic Preparation of Allium Sativum Abrogates OxLDL Mediated Atherogenic Events in Macrophages: An in Vitro and in Silico Approach. J. Ayurveda Integr. Med..

[51] Rath S., Jema J.P., Kesavan K., Mallick S., Pradhan J., Chainy G.B.N., Nayak D., Kaushik S., Dandapat J. (2024). Arsenic Album 30C Exhibits Crystalline Nano Structure of Arsenic Trioxide and Modulates Innate Immune Markers in Murine Macrophage Cell Lines. Sci. Rep..

[52] Ullman D. (2021). Exploring Possible Mechanisms of Hormesis and Homeopathy in the Light of Nanopharmacology and Ultra-High Dilutions. Dose-Response.

